# An Informatics Approach to Evaluating Combined Chemical Exposures from Consumer Products: A Case Study of Asthma-Associated Chemicals and Potential Endocrine Disruptors

**DOI:** 10.1289/ehp.1510529

**Published:** 2016-03-08

**Authors:** Henry A. Gabb, Catherine Blake

**Affiliations:** Graduate School of Library and Information Science, University of Illinois at Urbana-Champaign, Champaign, Illinois, USA

## Abstract

**Background:**

Simultaneous or sequential exposure to multiple environmental stressors can affect chemical toxicity. Cumulative risk assessments consider multiple stressors but it is impractical to test every chemical combination to which people are exposed. New methods are needed to prioritize chemical combinations based on their prevalence and possible health impacts.

**Objectives:**

We introduce an informatics approach that uses publicly available data to identify chemicals that co-occur in consumer products, which account for a significant proportion of overall chemical load.

**Methods:**

Fifty-five asthma-associated and endocrine disrupting chemicals (target chemicals) were selected. A database of 38,975 distinct consumer products and 32,231 distinct ingredient names was created from online sources, and PubChem and the Unified Medical Language System were used to resolve synonymous ingredient names. Synonymous ingredient names are different names for the same chemical (e.g., vitamin E and tocopherol).

**Results:**

Nearly one-third of the products (11,688 products, 30%) contained ≥ 1 target chemical and 5,229 products (13%) contained > 1. Of the 55 target chemicals, 31 (56%) appear in ≥ 1 product and 19 (35%) appear under more than one name. The most frequent three-way chemical combination (2-phenoxyethanol, methyl paraben, and ethyl paraben) appears in 1,059 products. Further work is needed to assess combined chemical exposures related to the use of multiple products.

**Conclusions:**

The informatics approach increased the number of products considered in a traditional analysis by two orders of magnitude, but missing/incomplete product labels can limit the effectiveness of this approach. Such an approach must resolve synonymy to ensure that chemicals of interest are not missed. Commonly occurring chemical combinations can be used to prioritize cumulative toxicology risk assessments.

**Citation:**

Gabb HA, Blake C. 2016. An informatics approach to evaluating combined chemical exposures from consumer products: a case study of asthma-associated chemicals and potential endocrine disruptors. Environ Health Perspect 124:1155–1165; http://dx.doi.org/10.1289/ehp.1510529

## Introduction

Much of the work in assessing risks associated with chemical exposure focuses on individual chemicals. However, communities face exposure from a variety of sources and the chemical load (also called body burden) is significantly higher than a century ago (Glegg and Richards 2007; Sanderson et al. 2013). More importantly, the dose response for chemical mixtures may be independent (additive), synergistic, or antagonistic (Sexton and Hattis 2007), and health outcomes can be influenced by both chemical and non-chemical stressors. With respect to chemicals, far-field exposure, such as persistent, high production volume industrial chemicals (Muir and Howard 2006), has been well explored, but near-field exposure from everyday consumer products such as shampoo, toothpaste, and makeup, account for a significant portion of our overall chemical load (Dodson et al. 2012; Egeghy et al. 2011; Koniecki et al. 2011).

In response to this increased awareness, risk assessments that once focused on a single pesticide or chemical (e.g., benzene, dioxin, and polychlorinated biphenyls) are moving towards a less-isolated and better-contextualized view of the multiple environmental agents to which humans are exposed (Jayjock et al. 2009). Cumulative risk assessments (CRA) consider multiple chemical and environmental stressors, though there is no single approach to measuring exposure (Choudhury et al. 2000; U.S. EPA 1986). The most challenging type of chemical mixtures to assess are the so-called coincidental mixtures that “occur by happenstance at a time or place of interest” (Sexton and Hattis 2007). It is not feasible to test every possible chemical mixture so new methods are needed to prioritize based on the level of human exposure (Dix et al. 2007; Sheldon and Cohen Hubal 2009), the nature of exposure, the severity of effects, and likelihood of interactions (Sexton and Hattis 2007).

Endocrine-disrupting compounds (EDCs), which are chemicals that may mimic hormones and alter endocrine signaling, are of particular interest because of their subtle and potentially far-reaching health effects (Colborn et al. 1993; Crisp et al. 1998; WHO/UNEP 2013), possibly including effects on oncogenesis (Soto and Sonnenschein 2010), metabolism (Elobeid and Allison 2008; Grün and Blumberg 2009; Heindel 2003; Newbold 2010; Newbold et al. 2008), and reproductive and nervous system development (Hengstler et al. 2011). Epidemiological studies have reported associations between prenatal exposure to chemicals classified as EDCs and early cognitive development (Engel et al. 2010; Factor-Litvak et al. 2014). In addition to potential health effects that may be subtle and difficult to observe, EDCs also have been associated with conditions like asthma. For example, some fragrance compounds may act as direct irritants to exacerbate and perhaps even cause asthma and other respiratory disorders (Bridges 2002; Kumar et al. 1995). In addition, there is evidence that some EDCs, including triclosan, glycol ethers, and phthalates can exacerbate asthma indirectly via immune sensitization (Anderson et al. 2013; Bornehag and Nanberg 2010; Bornehag et al. 2004; Choi et al. 2010).

Informatics approaches can contribute to the prioritization effort by integrating data from multiple sources (Jayjock et al. 2009; Sheldon and Cohen Hubal 2009). For example, the EPA’s NexGen risk assessment framework explored a range of methods including rapid screening to prioritize potentially harmful chemicals (Cohen Hubal et al. 2010; Collins et al. 2008; Cote et al. 2012; Dix et al. 2007; Egeghy et al. 2011; Krewski et al. 2014). Our goal is to help prioritize chemical combinations that should be further tested. To achieve this goal we introduce an informatics approach to identify combinations of chemicals in consumer products that are associated with asthma or have been identified as EDCs. The emphasis on such products is motivated in part by the frequency and type of exposure (consider products such as deodorant or toothpaste that are used every day and are applied directly to the skin or mucosa). In contrast to some environmental exposures where either community or regulatory pressure is needed to change exposure levels, individual consumers have more control over the products that they use, and hence their exposure levels. However, this control is not absolute. Some consumer products (e.g., vinyl shower curtains and pillow protectors, plastic storage containers) do not typically provide an ingredient list but may contain potentially harmful plasticizers (Dodson et al. 2012). When an ingredient list is provided, fragrance and flavoring chemicals are sometimes listed as generic fragrance or flavor. Fragrance and flavor mixtures can be designated trade secrets under the Fair Packaging and Labeling Act of 1967 (FPLA 1967) so their chemical composition need not be divulged. Also, plasticizers leached into a product from the container are not listed (Erythropel et al. 2014; Yang et al. 2011). Also, there may simply be a lack of safer alternative ingredients for consumers to choose. Finally, chemical synonymy, or different names referring to the same chemical, adds a layer of obfuscation that can hinder consumer identification of potentially harmful ingredients. Synonymy arises from the normal uncontrolled growth of language; in this case, the language describing chemical entities where trivial names represent the “convenient general language” of everyday chemistry, and systematic names represent the “legal language” (Tate 1967). Put another way, trivial names are simplified, common, or traditional chemical names that are not derived from a formal nomenclature while systematic nomenclatures attempt to unambiguously convey both the chemical entity and its chemical makeup (Leigh 2012). Chemicals can be listed on a product label using a systematic or trivial name. For example, methyl paraben is the trivial name of the common preservative chemical methyl 4-hydroxybenzoate (systematic name).

## Methods

### Select the Target EDC and Asthma-Associated Chemicals

The target chemicals for the present study were selected from a prior gas chromatography–mass spectrometry (GCMS) analysis of 213 consumer products to measure the levels of 55 potential EDC and asthma-associated chemicals (Dodson et al. 2012). They are listed in Table 1. These chemicals are not an exhaustive set of potential EDC or asthma-associated compounds, but they provide a basis of comparison between the informatics approach described in the present study and the prior GCMS analysis. A more complete set of potential EDCs can be found in the EDC DataBank (Montes-Grajales and Olivero-Verbel 2015), which incorporates the European Union and Endocrine Disruption Exchange lists of potential endocrine disruptors (http://eng.mst.dk/topics/chemicals/endocrine-disruptors/the-eu-list-of-potential-endocrine-disruptors/ and http://endocrinedisruption.org/endocrine-disruption/tedx-list-of-potential-endocrine-disruptors/overview).

**Table 1:** Prevalence of the target chemicals in consumer products and the degree of synonymy among consumer product ingredients.

Ingredient class	Chemical name	No. of products containing this chemical	No. of synonyms appearing in product ingredient lists	Synonyms (no. of products)
UV filter	Octinoxate	1,287	4	Octinoxate (556), octylmethoxycinnamate (30), octyl methoxycinnamate (46), ethylhexyl methoxycinnamate (655)
UV filter	Benzophenone-3	450	2	Oxybenzone (416), benzophenone-3 (34)
UV filter	Benzophenone-1	0
UV filter	Benzophenone	5	1	Benzophenone (5)
Cyclosiloxane	Dodecamethylcyclohexasiloxane	0
Cyclosiloxane	Decamethylcyclopentasiloxane	625	2	Decamethylcyclopentasiloxane (10), cyclomethicone (615)
Cyclosiloxane	Octamethylcyclotetrasiloxane	7	1	Octamethylcyclotetrasiloxane (7)
Glycol ether	2,2-Butoxyethoxyethanol	3	1	Butoxydiglycol (3)
Glycol ether	2,2-Methoxyethoxyethanol	0
Glycol ether	2-Phenoxyethanol	5,638	3	Phenoxyethanol (5,632), polyoxyethylene phenyl ether (1), 2 phenoxyethanol (5)
Glycol ether	2-Butoxyethanol	5	2	Butyl glycol (2), butoxyethanol (3)
Synthetic fragrance	Phenethyl alcohol	193	4	Phenethyl alcohol (180), phenylethyl alcohol (2), phenylethanol (6), phenyl ethyl alcohol (5)
Synthetic fragrance	Musk xylene	0
Synthetic fragrance	Musk ketone	0
Synthetic fragrance	Methyl ionone	197	4	Methyl ionone (6), alpha-isomethyl ionone (183), alpha-isomethylionone (5), methyl ionone gamma (3)
Synthetic fragrance	Isobornyl acetate	1	1	Bornyl acetate (1)
Synthetic fragrance	HHCB	0
Synthetic fragrance	DPMI	0
Synthetic fragrance	Diphenyl ether	1	1	Phenyl ether (1)
Synthetic fragrance	Bucinal	539	2	Lilial (71), butylphenyl methylpropional (468)
Synthetic fragrance	AHTN	1	1	Acetyl hexamethyl tetralin (1)
Natural fragrance	Terpineol	4	2	Terpineol (3), terpineol alpha (1)
Natural fragrance	Pinene	0
Natural fragrance	Methyl salicylate	105	3	Methyl salicylate (83), wintergreen oil (21), sweet birch oil (1)
Natural fragrance	Methyl eugenol	0
Natural fragrance	Linalool	2,517	2	Linalool (2,516), linalol (1)
Natural fragrance	Limonene	2,623	13	Limonene (2,334), d-limonene (17), limonen (1), orange flavor (44), lemon oil (83), lemon extract (15), sweet orange oil (4), orange oil (55), citrus limon oil (2), oil of lemon (2), orange flower oil (1), citrus sinensis oil (61), citrus sinensis peel oil (4)
Natural fragrance	Hexyl cinnemal	56	4	Hexyl cinnamic aldehyde (45), hexyl cinnamaldehyde (7), hexylcinnamaldehyde (3), alpha-hexylcinnamaldehyde (1)
Natural fragrance	Eugenol	429	1	Eugenol (429)
Natural fragrance	Benzylacetate	0
Alkylphenol	Nonylphenol diethoxylate	0
Alkylphenol	Nonylphenol monoethoxylate	0
Alkylphenol	4-t-Nonylphenol	0
Alkylphenol	Octylphenol diethoxylate	0
Alkylphenol	Octylphenol monoethoxylate	29	4	Octoxynol 9 (21), octoxynol-9 (3), octoxynol (1), octylphenoxypolyethoxyethanol (4)
Alkylphenol	4-t-Octylphenol	0
Ethanolamine	Diethanolamine	16	1	Diethanolamine (16)
Ethanolamine	Monoethanolamine	97	2	Ethanolamine (90), monoethanolamine (7)
Antimicrobial	Triclosan	104	1	Triclosan (104)
Antimicrobial	Triclocarban	12	1	Triclocarban (12)
Bisphenol A	Bisphenol A	0
Phthalate	Diethyl phthalate	5	1	Diethyl phthalate (5)
Phthalate	Di-n-propyl phthalate	0
Phthalate	Di-n-octyl phthalate	0
Phthalate	Di-n-hexyl phthalate	0
Phthalate	Di-n-butyl phthalate	26	1	Dibutyl phthalate (26)
Phthalate	Di-isononyl phthalate	0
Phthalate	Di-isobutyl phthalate	0
Phthalate	Di-cyclohexyl phthalate	0
Phthalate	Benzylbutyl phthalate	0
Phthalate	Bis(2-ethylhexyl) phthalate	0
Phthalate	Bis(2-ethylhexyl) adipate	29	2	Diethylhexyl adipate (25), dioctyl adipate (4)
Paraben	Butyl paraben	1,015	2	Butylparaben (1,008), butyl paraben (7)
Paraben	Ethyl paraben	1,364	3	Ethylparaben (1,356), ethyl paraben (6), catalase (2)
Paraben	Methyl paraben	4,510	3	Methylparaben (4,435), methyl paraben (74), methyl 4-hydroxybenzoate (1)
The variation in ingredient names highlights the need to take synonymy into account when searching ingredient lists for a particular chemical. The product totals should be considered a lower bound because some of the target chemicals are not always listed explicitly on a product label.				

### Create a Database of Consumer Products

The process used to create the database of consumer products is summarized here but greater methodological detail is provided in the “Supplemental Material (Database Methods).” Product names, ingredients, active ingredient concentrations, cost, brand, description, price, size, user directions, warnings and contraindications for 82,668 consumer products were retrieved from the online retail site, Drugstore.com, in April 2014. Only brand names, product names, and ingredients are used in the present analysis. Retrieval was done automatically using a robot scraper in compliance with the retailer’s terms of use and robot exclusion protocol (http://www.drugstore.com/robots.txt). The scraping program was written in Java and used the XPath extensions to traverse the retailer’s published site map, and the Apache HttpClient (version 3.1; Apache Software Foundation) to request product web pages. (However, users should be aware that HttpClient is no longer supported. Its functionality has been incorporated into Apache HttpComponents so new development should use this package or some other supported HTTP client.) Ingredient lists were extracted from the raw HTML and parsed into individual ingredients using Python (version 2.7; Python Software Foundation) and regular expressions. Briefly, each ingredient list was converted to lowercase and extraneous, non-ingredient text such as “may contain” or “certified organic” was removed. Parenthetical text was retained because it often contains useful information such as alternative names that can help identify an ingredient. Active concentrations were saved but not used because the present analysis is only concerned with the presence or absence of the target chemicals in consumer products. Parsing the ingredient lists yielded 663,075 product–ingredient combinations, though many ingredients appear in multiple products under multiple names. For example, water appears in 19,000 products and may be listed as purified water, aqua, agua, eau, etc.

Given the size of the dataset, it is infeasible to examine every datum for correctness. Therefore, frequent spot checks of random samples were used to validate and refine each stage of data processing. However, further validation was performed before the final tabulation of results. Validation of brand and product names was performed by manual inspection of 100 randomly selected products to confirm that the necessary data was correctly extracted from the raw HTML. Accuracy was 100% (i.e., every brand and product name in the sample was correct). Processing of the ingredient strings was validated by randomly selecting 100 products for manual inspection. Parsed ingredient lists were compared to the raw ingredient strings to confirm that ingredient names and accompanying parenthetical text are correctly extracted. Of the 1,587 ingredients in this sample, 1,547 (97%) were correctly extracted. Of the 40 incorrectly extracted ingredients, 24 were slash-delimited polymers, fatty acids, or mixtures (e.g., styrene/acrylates copolymer, acrylates/c10 30 alkyl acrylate crosspolymer, cetyl peg/ppg-10/1 dimethicone, caprylic/capric triglyceride, pvm/ma copolymer). Ingredient string parsing was not modified to handle these types of ingredients because they are not the focus of the present analysis and because it is unclear how they should be parsed. Missing commas in the ingredient list caused the remaining 16 incorrectly parsed ingredients. The “Supplemental Material (Database Methods)” contains more information about brand and product name extraction, ingredient string parsing, and validation.

### Unify Ingredient Names

PubChem and the Unified Medical Language System (UMLS) were used to unify synonymous ingredient names. PubChem was launched in 2004 as a repository of information about the biological activity of small molecules. It is hosted by the National Center for Biotechnology Information (NCBI). “The primary aim of PubChem is to provide a public on-line resource of comprehensive information on the biological activities of small molecules accessible to molecular biologists as well as computation and medicinal chemists” (Bolton et al. 2008). It consists of three distinct, community-supported databases: PubChem Substance, PubChem Compound, and PubChem BioAssay that are interlinked through substance, compound, and assay identifiers. Users contribute and validate data but the actual PubChem database processing is highly automated and there is little manual curation or central control of input by the NCBI (Bolton et al. 2008).

The PubChem Compound (Kim et al. 2016) database is most appropriate for our purposes (i.e., matching product ingredient names to chemical identifiers) because its chemical synonym list is large and it generally maps chemicals to Chemical Abstracts Service Registry Numbers (CAS-RN) and IUPAC International Chemical Identifiers (InChI). It also maps chemicals to Medical Subject Headings (MeSH) to facilitate integration with PubMed and the UMLS. The list of synonyms for each Compound Identifier (CID) was downloaded from PubChem in December 2014. This file contained approximately 39 million CIDs and 150 million synonyms. Some preprocessing was required to optimize name matching. Our transformations are similar to those applied to other chemical dictionaries and chemistry text processing applications (Hettne et al. 2009; McCray et al. 2001; Rogers and Aronson 2008; Schwartz and Hearst 2003). First, each synonym is converted to lowercase. Second, the long and abbreviated forms of a synonym [e.g., “acetyl hexamethyl tetralin (ahtn)”] are separated. Third, syntactic inversion is performed on synonyms that contain a comma followed by a space. For example, acetyl hexamethyl tetralin has a synonym “ethanone, 1-(5,6,7,8-tetrahydro-3,5,5,6,8,8-hexamethyl-2-naphthalenyl)-” that is inverted to yield an additional synonym “1-(5,6,7,8-tetrahydro-3,5,5,6,8,8-hexamethyl-2-naphthalenyl)-ethanone.” Finally, each synonym is split on whitespace to obtain a list of terms that are matched to product ingredient names. For example, acetyl hexamethyl tetralin is a three-term synonym that would be matched to three-term ingredient names, whereas ahtn would be matched to one-term ingredient names.

The UMLS project began in 1986 at the National Library of Medicine and the first version was released in 1989 (Humphreys and Lindberg 1993; Humphreys et al. 1998). The UMLS is composed of three components, the SPECIALIST lexicon, semantic network, and a metathesaurus that aligns the content of 170 different independently maintained controlled vocabularies covering many aspects of biomedicine (e.g., diseases, drugs and chemicals, surgical procedures, literature indexing, medical billing). A controlled vocabulary is a curated list of terms that represent the important concepts of a particular field. The terms in these vocabularies are mapped to Concept Unique Identifiers (CUI). The UMLS was downloaded from http://www.nlm.nih.gov/research/umls in December 2014. Fifteen vocabularies were included in our installation and the number of terms in each vocabulary gives its relative contribution to our UMLS installation (Table 2). The strings associated with each concept undergo preprocessing similar to that described by Hettne et al. (2010) to obtain a list of terms that are matched to product ingredient names.

**Table 2:** UMLS vocabularies used in this study.

Vocabulary	No. of terms	Official name
AOD	20,685	Alcohol and Other Drug Thesaurus
CHV	146,324	Consumer Health Vocabulary
DXP	10,113	DXplain (an expert diagnosis program)
MSH	815,608	Medical Subject Headings
MTH	171,407	UMLS Metathesaurus
MTHFDA	86,069	Metathesaurus FDA National Drug Code Directory
MTHSPL	113,248	Metathesaurus FDA Structured Product Labels
NCBI	1,265,703	National Center for Biotechnology Information Taxonomy
NCI	255,108	National Cancer Institute Thesaurus
RXNORM	628,521	RxNorm Vocabulary
SNM	44,274	Systemized Nomenclature of Medicine
SNMI	164,179	Systemized Nomenclature of Human and Veterinary Medicine
SNOMEDCT_US	1,225,189	Systemized Nomenclature of Medicine—Clinical Terms (U.S. Edition)
SNOMEDCT_VET	89,572	Veterinary Extension to SNOMED-CT
SRC	1,018	Metathesaurus Source Terminology Names
A vocabulary is a curated list of terms that represent the important concepts of a particular field. The number of terms in each vocabulary gives its relative contribution to the UMLS installation.		

Synonyms must resolve to the same identifier if they are to be useful. In the UMLS, this identifier is the CUI. For example, searching the UMLS for octinoxate, octyl methoxycinnamate, octyl methoxycinnamate, or ethylhexyl methoxycinnamate will return the same CUI (C0046100). Searching the UMLS for C0046100 will return octinoxate and all of its synonyms. PubChem performs the same function but refers to its unique identifiers as CIDs. Octinoxate, octylmethoxycinnamate, octyl methoxycinnamate, and ethylhexyl methoxycinnamate all have the same CID (5355130). Searching PubChem for 5355130 will return octinoxate and all of its synonyms. We combine PubChem and the UMLS to get greater coverage of the chemical namespace.

### Match Ingredient Names to PubChem and the UMLS

We used a dictionary-based, exact-matching approach to map ingredient names to terms in PubChem or the UMLS. As described above, product ingredients, PubChem synonyms, and UMLS concepts were parsed into terms. For example, the ingredient, methylparaben, is a single term but its synonym, methyl paraben, consists of two terms: methyl and paraben. One-term ingredients are simply compared to one-term PubChem synonyms and one-term UMLS concepts, two-term ingredients are compared to two-term synonyms/concepts, etc. looking for exact matches. If a match is found the ingredient is mapped to the CID and/or CUI. In this way, synonymous ingredient names are mapped to the same CID and/or CUI. For example, methyl paraben is mapped to a single CID and/or CUI whether it appears in a product label as methyl paraben, methylparaben, or methyl 4-hydroxybenzoate. This is absolutely necessary to get accurate counts of ingredients and the products containing those ingredients, as our results will demonstrate.

Exact term-by-term matching was used for three reasons. First, systematic names are rare in consumer product ingredient lists so complex parsing based on chemical morphology (Leaman et al. 2015; Lowe et al. 2011) is unnecessary. Trivial names are easily parsed into terms that can be matched exactly. Second, PubChem and UMLS entries often have dozens, sometimes hundreds, of synonyms, so a trivial name appearing in a product ingredient list is likely to be among those synonyms. Third, sophisticated string matching techniques (e.g., Dice’s coefficient, edit distance, and Levenshtein ratio) (Dice 1945; Navarro 2001) are prone to false positives and false negatives when dealing with chemical names. [The “Supplemental Material (Database Methods)” contains more information about the application of these string matching methods.] For example, “vitamin a” and “vitamin e” are similar strings but different chemicals (false positive), whereas “dimethyl ether” and “methoxymethane” are dissimilar strings but the same chemical (false negative). A dictionary-based approach using exact matching is therefore the best method to map an ingredient name to a chemical identifier.

### Account for Homonymy in Chemical Identifiers

**Figure 1:**
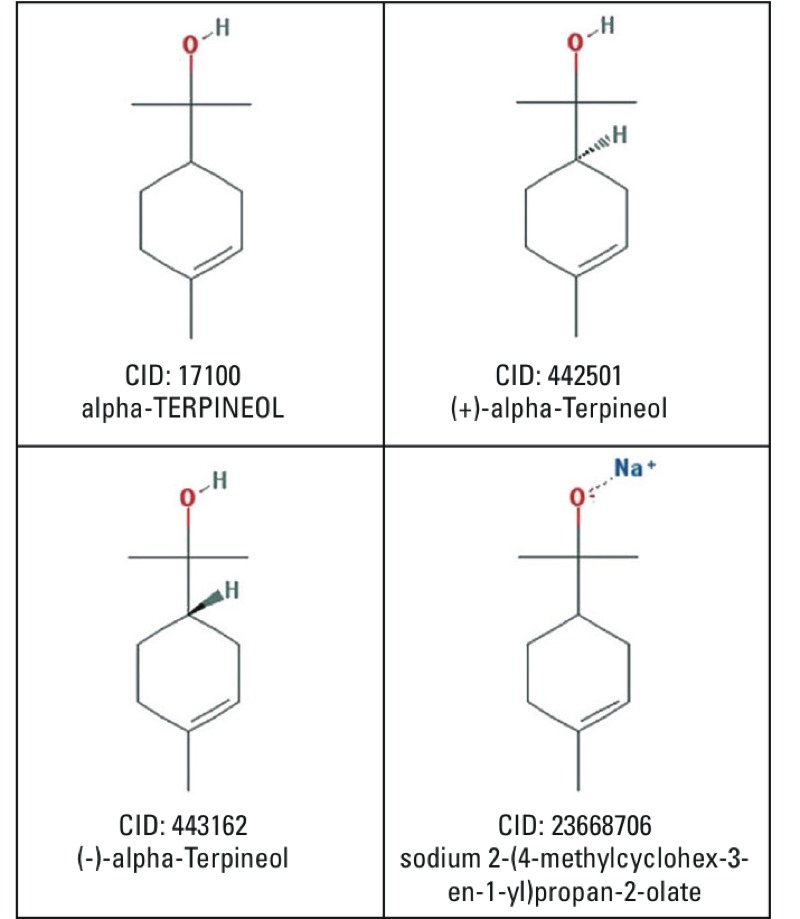
Example of homonymy in chemical naming. Chemical homonymy occurs when the same name can refer to different chemicals. Terpineol, its stereoisomers, and its sodium salt each have a different CID in PubChem but share common synonyms. Therefore, the same chemical name can match more than one PubChem CID. These images were taken from PubChem ( Kim et al. 2016).

Chemical synonymy, as defined previously, occurs when different names refer to the same chemical (e.g., vitamin E and tocopherol). Chemical homonymy occurs when the same name can refer to different chemicals [e.g., the generic name Terpineol can refer to various stereoisomers or salts of the parent compound, 2-(4-methylcyclohex-3-en-1-yl)propan-2-ol]. The degeneracy of two-dimensional molecular descriptors (i.e., different compounds sharing the same descriptor) is a known problem in chemistry (Faulon et al. 2005; Randić 1984). Similarly, shared synonyms among the various salts and stereoisomers of a compound can lead to homonymy among PubChem CIDs (Figure 1). The UMLS comprises multiple vocabularies (Table 2) so the same chemical name can map to different concepts depending on context, though the degree of homonymy among UMLS CUIs is significantly less than PubChem CIDs. Thus, a chemical name (or in this study the ingredient name) can refer to more than one CID or CUI. However, this also means that when searching for a particular chemical among tens of thousands of consumer product ingredient lists, all the PubChem or UMLS synonyms associated with that chemical plus the synonyms associated with its homonymic CIDs or CUIs are available for possible matching.

To account for homonymy, synonyms for a given chemical are compared to the synonyms of every other chemical in PubChem. If a match is found, the two CIDs are considered to be homonymic. Fourteen of the 55 target chemicals had at least one homonymic CID (Table 3). For example, the synthetic fragrance, methyl ionone (CID: 5371084), shares synonyms with two other chemicals: alpha-Cetone (CID: 5372174) and 127-42-4 (CID: 16751505). The latter is a CAS-RN that is listed among the synonyms of both CIDs. In order to maximize coverage, the synonyms associated with all three CIDs are used when looking for methyl ionone among the consumer product ingredient lists.

**Table 3:** Homonymy of PubChem CIDs.

CID	Chemical name	No. of synonyms	No. of homonymic CIDs	No. of synonyms taking homonymic CIDs into account
5355130	Octinoxate	88	3	99
8572	Benzophenone-1	107	1	109
5371084	Methyl ionone	64	2	116
6448	Isobornyl acetate	91	10	234
17100	Terpineol	119	3	191
6549	Linalool	118	2	197
22311	Limonene	253	2	407
7585	alpha-Hexylcinnamaldehyde	25	2	111
107	Benzylacetate	170	1	215
5590	4-tert-Octylphenol monoethoxylate	193	1	198
6623	Bisphenol A	189	1	204
2347	Benzyl butyl phthalate	117	1	119
8343	Bis(2-ethylhexyl) phthalate	179	1	182
7184	Butyl paraben	141	1	145
Fourteen of the 55 chemicals listed by Dodson et al. (2012) had at least one homonymic CID. In some cases, this significantly increased the number of potential synonyms associated with the chemical name. For example, accounting for homonymy increases the number of alpha-hexylcinnamaldehyde synonyms from 25 to 111.				

### Assign Product Categories

We used an approach similar to Goldsmith et al. (2014) to annotate product categories. Product pages on retail sites typically include the product’s location in the retailer’s hierarchy. For example, toothpaste might be in the home→personal care→oral care→toothpaste branch of the retail hierarchy. This information is included to help customers navigate the retail site more efficiently. We use it to categorize products because retail categories are objective and retailers have a vested interest in making sure they are correct. For our purposes, the most specific level of the retail branch (toothpaste in the example above) that maps to one of our categories is used to assign the product category.

The categories and sample sizes in our database are shown in Table 4. As much as possible, we tried to map the products in our database to one of the categories used in Dodson et al. (2012). Five of their categories (cat litter, pillow protectors, vinyl shower curtains, car interior cleaners, and car air fresheners) were excluded because our database does not contain any representative products. We also combined their household cleaning categories (i.e., surface, floor, tub and tile, and glass cleaners and scrubbing powder) into a single category (i.e., cleaner) because the sample sizes of the specific categories are small relative to the other household categories in Table 4. Combining them into a single category helps to balance sample sizes within our broad household category. Finally, we added several categories (mostly under medication and diet) for products that were in our database but were not tested by Dodson et al. (2012).

**Table 4:** Product categories, sample sizes, the percentage of products in each category that contain at least one of the target chemicals, and the number of target chemicals appearing in each product category.

Broad category	Specific category	No. of products	Percentage containing one or more target chemicals	No. of target chemicals in category
Household	Air fresheners	197	15.3	4
	Cleaner	108	5.5	3
	Diapers	72	2.1	1
	Dishwashing	121	14.2	7
	Laundry	273	3.3	6
	Pesticide	158	10.0	7
	Pet supplies	612	2.1	3
	Other	395	5.7	9
Personal cleaning	Bar soap	620	6.3	11
	Body wash	1,075	33.4	18
	Facial cleanser	622	57.5	19
	Hand sanitizer	44	11.3	4
	Liquid soap	289	29.7	9
	Other	501	44.0	10
Personal care	Body oil & body spray	231	28.2	12
	Deodorant & antiperspirant	518	12.3	13
	Feminine hygiene	237	23.1	8
	Lotion & moisturizer	2,467	66.5	19
	Sexual health	333	23.6	7
	Shaving & hair removal	480	34.3	16
	Sunscreen	503	71.8	14
	Other	1,094	51.6	19
Oral care	Mouthwash	154	24.7	3
	Toothpaste	332	12.8	9
Hair care	Conditioner	1,363	58.4	20
	Hair color	256	48.9	10
	Hair styling	1,479	63.3	18
	Shampoo	1,338	43.9	19
	Other	53	48.3	11
Cosmetics	Bronzers & tanners	189	69.3	13
	Eye makeup	1,688	66.8	15
	Foundation	1,657	72.3	14
	Fragrance & perfume	505	51.4	12
	Lip makeup	1,606	42.3	13
	Manicure & pedicure	1,792	14.9	22
	Other	243	62.6	13
Medication	Oral medication	1,957	7.3	13
	Topical medication	772	25.8	14
	Other	360	10.0	6
>Diet	Food	3,324	0.8	2
	Supplements	4,291	1.2	6
	Tea	610	3.1	1
	Vitamins	3,583	0.9	4
Other	Other	473	14.9	12

Assigning a category to a product is usually straightforward but some products can exist in more than one category (e.g., products labeled as “shampoo and conditioner” or “shampoo and body wash”). Therefore, the most specific level of the retail hierarchy that matches one of our categories is used to make the assignment. This approach worked well. Only 67 (0.2%) out of 38,975 products were assigned to more than one category. Products are assigned to “other” when their broad and/or specific category cannot be determined. Only 3,119 (8%) products could not be assigned a category. Final category assignments were validated using a random sample of 100 products. Accuracy was high (96%). Of the four incorrectly categorized products, one was due to an error in the retail hierarchy; specifically, an eyeliner product was incorrectly placed in the lip liner branch of the hierarchy. The rest were due to ambiguities in category mapping. For example, one of the incorrect assignments was a topical medication in a relatively sparse branch of the retail hierarchy: medicine & health→pain & fever relief→shop by active ingredient→natural ingredients. The most specific level of the retail hierarchy that maps to one of our product categories is “pain & fever relief” so it was used to make the assignment. In our categorization scheme, “pain & fever relief” maps to oral medications because most products in this category are oral medications. The “Supplemental Material (Database Methods)” contains more information about category assignment and its validation.

## Results

### Consumer Product Database

The database contains 41,277 products that have at least one ingredient listed on the product label. Exact duplicates (the same brand and product name scraped from different locations) and partial duplicates (different sizes of the same product) were pruned to avoid inflating ingredient counts. [The “Supplemental Material (Database Methods)” contains more information about the removal of duplicate products.] The final database comprises 38,975 distinct products (from 8,099 brand names). The database contained 32,231 distinct ingredient names after removal of duplicates. We were able to map 7,486 ingredients to a CID and/or CUI after resolving synonymous names (e.g., water, eau, agua, distilled water, purified water, etc.). This is much larger than the 8,921 products with 1,797 unique chemicals found in a database of consumer product ingredients that was recently constructed by scraping Material Safety Data Sheets (MSDS) (Goldsmith et al. 2014). In contrast to MSDS, that are only required to list those ingredients known to be hazardous, the database used here includes all ingredients listed on a product label.

Two other consumer products databases are similar to this work: Skin Deep (http://www.ewg.org/skindeep/), which was created by the Environmental Working Group, and the Household Products Database (http://householdproducts.nlm.nih.gov/). We created our own database because neither of these resources is downloadable or otherwise amenable to bulk querying or integration with other data sources. Another EDC database, the EDCs DataBank (http://edcs.unicartagena.edu.co), was published after the present analysis was completed (Montes-Grajales and Olivero-Verbel 2015). It focuses primarily on structural chemistry but it also provides some data on EDC occurrence within broad product categories so it will likely be a useful resource for future EDC research.

### Prevalence of Potentially Harmful Chemicals in Consumer Products

**Figure 2:**
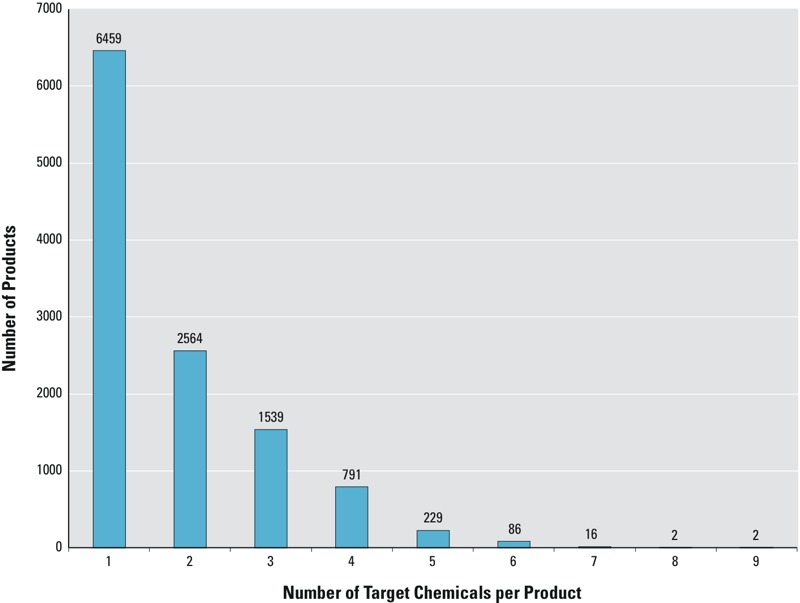
Of the 38,975 consumer products in our sample, 11,688 (30%) contain at least one of the potentially harmful chemicals identified in Dodson et al. (2012): 6,459 contain only one target chemical, 2,564 contain two, 1,539 contain three, etc. Of the 11,688 products that contain a target chemical, 6,459 (55%) contain only one, while 5,229 (45%) contain more than one.

**Figure 3:**
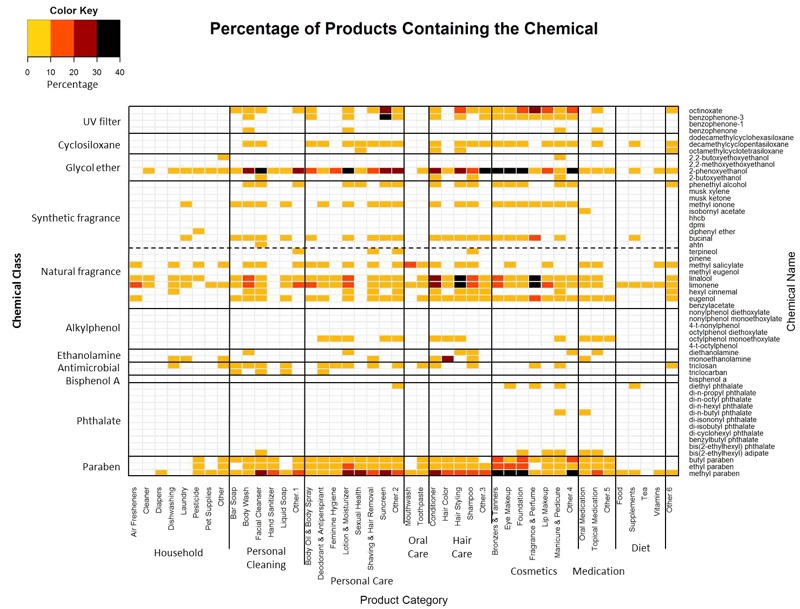
Heat map showing chemical prevalence by product category. Broad and specific consumer product categories are shown along the horizontal axis. Chemical class is shown on the left vertical axis and specific chemical ingredients are shown on the right vertical axis. White indicates that a chemical was not found in a product category. Yellow indicates that > 0–10% of the products in the category contain the chemical. Orange indicates that > 10–20% of the products contain the chemical. Dark red indicates that > 20–30% of the products contain the chemical. Black indicates that > 30–40% of the products contain the chemical.

The EDC and asthma-associated chemicals targeted by Dodson et al. (2012) are prevalent in consumer products; particularly among cosmetics, hair care, and personal care products. Table 4 shows the prevalence by product category. Table 1 shows the prevalence by target chemical. Of the 38,975 consumer products in our sample, 11,688 (30%) contain at least one of the target chemicals. Of those, 6,459 (55%) contain only one while 5,229 (45%) contain more than one (Figure 2). The percentage of products in each category that contain a given chemical is shown in Figure 3. The most common target chemicals and product hotspots are readily apparent (Figure 3). Phenoxyethanol (a glycol ether and common preservative) is the most frequently occurring target chemical, followed by methyl paraben (another common preservative), the natural fragrances limonene and linalool, and octinoxate [an ultraviolet (UV) filter]. These chemicals span many product categories.

Cosmetics and hair care products have several hotspots for glycol ethers, fragrances, parabens, and to a lesser extent, UV filters (Figure 3). It is not surprising that UV filters are common in sunscreens and some cosmetics and hair care products. However, this supports the validity of our parsing and matching process, especially given the number of synonyms for these chemicals that appear in consumer product labels (Table 1).

The antimicrobials, triclosan and triclocarban, do occur in our sample but they are relatively rare (Table 1, Figure 3), which is not surprising as these chemicals are being phased out of consumer products due to increasing consumer pressure (APUA 2011; Coleman-Lochner et al. 2014; EWG 2014) and EPA scrutiny (U.S. EPA 2010, 2015). Personal care, hair care, and cosmetic products have hotspots for glycol ethers, natural fragrances, and parabens (Figure 3).

“Fragrance” is the second most common ingredient in our product sample after water. Various flavors and flavorings also occur frequently. While the target chemicals limonene, linalool, and a few other natural fragrances are fairly common among products in our sample, the synthetic fragrance chemicals are comparatively rare (Table 1, Figure 3).

### Co-Occurrence among the Target Chemicals

As mentioned above, 5,229 products in the database contain more than one of the target chemicals (Figure 2). The 20 two- and three-way target chemical combinations that were most likely to appear in the same product are listed in Tables 5 and 6, respectively, and complete lists of all two- and three-way combinations are provided in Excel File Tables S1 and S2. (For complete lists of four-, five- and six-way combinations, see Excel File Tables S3, S4, and S5, respectively. This dataset can also be examined interactively at https://uiuc-gslis-blake.shinyapps.io/App-ChemComboBrowser.) The product totals given in these tables should be considered a lower bound because previous GCMS analysis detected the target chemicals in products where manufacturers either did not provide an ingredient list or specified “fragrance” or “flavor” instead of listing the precise ingredients in these mixtures (Dodson et al. 2012). All of the target chemicals except the ethanolamines have been implicated in endocrine disruption (Dodson et al. 2012). The phthalates, fragrances, glycol ethers, and antimicrobials have also been implicated in the frequency and severity of asthma attacks (Anderson et al. 2013; Bornehag and Nanberg 2010; Bornehag et al. 2004; Bridges 2002; Choi et al. 2010; Dodson et al. 2012).

**Table 5:** Twenty most frequently occurring pairs among the target chemicals.

Chemical 1	Chemical 2	No. of products containing this pair
Methyl paraben (PB)	2-Phenoxyethanol (GE)	1,872
Linalool (NF)	Limonene (NF)	1,850
Methyl paraben (PB)	Ethyl paraben (PB)	1,329
Ethyl paraben (PB)	2-Phenoxyethanol (GE)	1,081
Butyl paraben (PB)	Methyl paraben (PB)	889
Linalool (NF)	2-Phenoxyethanol (GE)	797
Limonene (NF)	2-Phenoxyethanol (GE)	780
Butyl paraben (PB)	2-Phenoxyethanol (GE)	684
Butyl paraben (PB)	Ethyl paraben (PB)	595
Linalool (NF)	Methyl paraben (PB)	481
Methyl paraben (PB)	Limonene (NF)	427
Eugenol (NF)	Linalool (NF)	362
Eugenol (NF)	Limonene (NF)	302
Linalool (NF)	Ethyl paraben (PB)	179
Ethyl paraben (PB)	Limonene (NF)	155
Benzophenone-3 (UV)	Methyl paraben (PB)	140
Eugenol (NF)	2-Phenoxyethanol (GE)	132
Llinalool (NF)	Butyl paraben (PB)	131
Benzophenone-3 (UV)	2-Phenoxyethanol (GE)	122
Butyl paraben (PB)	Limonene (NF)	113
The chemical classes are as follows: methyl, ethyl, and butyl paraben comprise the parabens (PB); linalool, limonene, and eugenol comprise the natural fragrances (NF); and glycol ethers (GE) and UV filters (UV) are represented by 2-phenoxyethanol and benzophenone-3, respectively. A complete list of two-way combinations is provided in Excel File Table S1. The product totals should be considered a lower bound because some of the target chemicals are not always listed explicitly on a product label.		

**Table 6:** Twenty most frequently occurring three-way combinations of the target chemicals.

Chemical 1	Chemical 2	Chemical 3	No. of products containing this ternary combination
Methyl paraben (PB)	Ethyl paraben (PB)	2-Phenoxyethanol (GE)	1,059
Butyl paraben (PB)	Methyl paraben (PB)	2-Phenoxyethanol (GE)	662
Butyl paraben (PB)	Methyl paraben (PB)	Ethyl paraben (PB)	587
Linalool (NF)	Limonene (NF)	2-Phenoxyethanol (GE)	566
Butyl paraben (PB)	Ethyl paraben (PB)	2-Phenoxyethanol (GE)	530
Linalool (NF)	Methyl paraben (PB)	Limonene (NF)	308
Eugenol (NF)	Linalool (NF)	Limonene (NF)	272
Linalool (NF)	Methyl paraben (PB)	2-Phenoxyethanol (GE)	241
Methyl paraben (PB)	Limonene (NF)	2-Phenoxyethanol (GE)	216
Linalool (NF)	Methyl paraben (PB)	Ethyl paraben (PB)	176
Methyl paraben (PB)	Ethyl paraben (PB)	Limonene (NF)	153
Linalool (NF)	Ethyl paraben (PB)	2-Phenoxyethanol (GE)	150
Ethyl paraben (PB)	Limonene (NF)	2-Phenoxyethanol (GE)	131
Linalool (NF)	Ethyl paraben (PB)	Limonene (NF)	123
Linalool (NF)	Butyl paraben (PB)	Methyl paraben (PB)	122
Linalool (NF)	Butyl paraben (PB)	Ethyl paraben (PB)	106
Linalool (NF)	Butyl paraben (PB)	2-Phenoxyethanol (GE)	103
Butyl paraben (PB)	Methyl paraben (PB)	Limonene (NF)	102
Eugenol (NF)	Linalool (NF)	2-Phenoxyethanol (GE)	95
Butyl paraben (PB)	Limonene (NF)	2-Phenoxyethanol (GE)	91
The chemical classes are as follows: methyl, ethyl, and butyl paraben comprise the parabens (PB); linalool, limonene, and eugenol comprise the natural fragrances (NF); and glycol ethers (GE) are represented by 2-phenoxyethanol. A complete list of three-way combinations is provided in Excel File Table S2. The product totals should be considered a lower bound because some of the target chemicals are not always listed explicitly on a product label.			

Examining the most common chemical pairs (Table 5) by chemical class indicates that the parabens and glycol ethers (in this case, 2-phenoxyethanol) co-occur 3,637 times in our database. (Note that the chemical combinations in Tables 5 and 6 are not mutually exclusive. For example, a methyl paraben/2-phenoxyethanol pair does not preclude a methyl paraben/ethyl paraben pair in the same product.) Natural fragrance pairs occur 2,514 times. Glycol ethers and natural fragrances co-occur 1,709 times. Parabens and natural fragrances co-occur 1,486 times. Glycol ethers and UV filters (in this case, benzophenone-3) co-occur 122 times. Of the 2,665 products that contain at least three of the target chemicals, the same pairs of chemical classes (except glycol ether/UV filter) are present among the most common three-way chemical combinations: paraben/glycol ether (2,251 times), paraben/natural fragrance (1,090 times), and natural fragrance/glycol ether (661 times). However, natural fragrance, paraben, and glycol ether chemicals are also frequently combined (932 times). The same chemical classes dominate the four- to six-way combinations (i.e., parabens, glycol ethers, and natural fragrances). However, UV filters and synthetic fragrances also begin to appear, though they are not as frequent (see Excel File Tables S1–S5 for all two- to six-way chemical combinations in the dataset). These combinations have the potential to simultaneously affect endocrine function and asthma severity. Excel File Tables S1–S5 can be used to prioritize which chemical combinations should be evaluated using traditional means to establish whether their cumulative toxicity is independent (additive), synergistic, or antagonistic. (The combinatorial data can be examined interactively at https://uiuc-gslis-blake.shinyapps.io/App-ChemComboBrowser.)

### Chemical Synonymy

Just over half (31 out of 55) of the EDC and asthma-associated chemicals targeted in this study appear among the 38,975 consumer products (Table 1). Of these, 19 appear under more than one name. Therefore, synonymy must be taken into account in order to get an accurate count of products containing a particular ingredient. For example, bucinal is a fairly common synthetic fragrance but simply searching ingredient lists for bucinal will miss all 539 products containing this chemical. Searching for its synonym, lilial (71 products), will still miss most of the products containing this chemical because it is more commonly listed as butylphenyl methylpropional (468 products). It is not intuitively obvious, even to a chemist, that bucinal, lilial, and butylphenyl methylpropional are synonyms. A lay consumer is unlikely to recognize chemical synonyms. Such is the case with many of the chemicals listed in Table 1, e.g.: octinoxate, benzophenone-3, decamethylcyclopentasiloxane, methyl salicylate, limonene, and 4-tert-octylphenol monoethoxylate. Methyl salicylate and limonene further illustrate the gap between chemical names and ingredient labels. Although the chemical names are used most often, marketing factors may motivate the use of natural sounding names such as wintergreen oil or sweet birch oil instead of the chemical equivalent methyl salicylate.

## Discussion

The present study applies an informatics approach to the analysis of EDC and asthma-associated chemicals in everyday consumer products. We evaluated the prevalence of 55 chemicals from a similar set of product categories as a recent GCMS analysis (Dodson et al. 2012) and found that these target chemicals are common among the 38,975 products in the database (Tables 1 and 4, Figure 3), which is further evidence that everyday consumer products may contribute to near-field exposure. The advantage of an informatics approach is in the number of products that can be considered. The cost and labor involved in GCMS make it impractical to analyze the nearly 40,000 products in our database. In contrast, the traditional approach tested 213 different products in 42 composite samples (Dodson et al. 2012). The present study found products with target chemicals that are not detected in the small GCMS sample. For example, our results show that toothpastes contain the same three target chemicals found in the GCMS analysis: the antimicrobial triclosan and the natural fragrances methyl salicylate and eugenol. However, several more of the target chemicals also appear in toothpaste ingredient lists: phenoxyethanol, linalool, limonene, butyl paraben, ethyl paraben, and methyl paraben (Figure 3). The antimicrobials further demonstrate the utility of the database approach. We detect triclocarban in four product categories (bar soap, facial cleanser, liquid soap, and deodorant and antiperspirant) (Figure 3) whereas it is only detected in one GCMS sample (bar soap). Our sample contains triclosan in 17 product categories (Figure 3) compared to only three of the GCMS samples. Finally, Dodson et al. (2012) only analyzed six product categories for UV filters (sunscreen and shaving cream) and cyclosiloxanes (sunscreen and car interior cleaners). By comparison, the database contains UV filters and cyclosiloxanes in 22 product categories (Figure 3).

In addition to larger product sample size, the informatics approach can also consider a larger number of target chemicals. The present analysis looked at 55 previously studied EDC and asthma-associated chemicals. However, expanding the number of targets to hundreds or even thousands of chemicals, as envisioned by the Tox21 consortium (U.S. EPA 2008), is straightforward because the underlying database structure and SQL (Structured Query Language) queries, which are small by modern database standards, remain the same. Only the table of target chemicals would be changed to include more targets. The only caveat is that the target chemicals must be represented in PubChem or the UMLS. PubChem and the UMLS already contain tens of millions of chemicals and continue to grow, so toxicologically interesting chemicals are likely to be represented.

However, the informatics approach also has limitations. First, the product and ingredient lists must be made readily available. For example, the car interior cleaners that were analyzed in the prior study were not in the websites that we scraped. Also, many of the products in our database do not typically provide an ingredient list (e.g., vinyl shower curtains and plastic storage containers). The second, and more important, limitation is that product manufacturers are not required to specify every chemical in the ingredient list. The FPLA (1967) requires manufacturers to list ingredients in “descending order of predominance” but it does not require them to disclose trade secrets. The complex mixtures of natural and synthetic fragrances and flavorings that go into many consumer products are often treated as trade secrets that are not subject to precise ingredient labeling. They are simply listed as fragrance or flavor on the product label. This highlights the main advantage of GCMS, which can detect chemicals that do not appear in an ingredient list. For example, bisphenol A does not appear in any of the ingredient lists in our product sample but its presence was detected in products from several categories, including those that do not normally provide an ingredient list like vinyl shower curtains or pillow protectors (Dodson et al. 2012). The GCMS analysis also detects more phthalates than appear in our database. With the exception of a few cosmetics categories, particularly nail polish (manicure & pedicure), phthalates are uncommon among the products in our sample (Table 1, Figure 3).

Analysis of consumer product ingredient lists illustrates how chemical synonymy can hinder consumer decision-making with respect to the chemicals in their products. For example, consumers trying to manage their asthma read a news article claiming that a specific fragrance chemical may exacerbate asthma attacks. They check the ingredient lists on the products in their homes and feel satisfied that none of them contain the fragrance. This is a false sense of security unless they have also checked for commonly used synonyms for the fragrance that may not have been mentioned in the news source. This same scenario can be applied to many other chemical ingredients, as illustrated in Table 1. Apply the reverse logic to a consumer looking for a fragrance-free product. Many products only specify “fragrance” (the second most common ingredient after water) on the ingredient label instead of listing each fragrance chemical in the mixture. These products are easy to avoid. Ironically, products that explicitly list fragrance chemicals may be harder for a consumer to assess. Consider a product that lists butylphenyl methylpropional but not fragrance in the ingredient label. Unless consumers know that butylphenyl methylpropional is a fragrance chemical, they may mistakenly assume that the product is fragrance free. Risk perception adds another dimension to the problem of chemical synonymy. Namely, consumers may choose a product that lists wintergreen oil as an ingredient instead of one that lists methyl salicylate because the product with wintergreen oil seems more “natural,” in spite of the fact that wintergreen oil and methyl salicylate are synonymous in PubChem.

As mentioned previously, cumulative risk assessments consider multiple stressors but performing risk assessment on all possible chemical mixtures is infeasible. The informatics approach described here can help prioritize testing based on the likelihood of co-exposure. In addition to individual ingredient prevalence (Table 1, Figure 3), it is also possible to determine the most likely chemical combinations within a large sample of consumer products (Tables 5 and 6). It is not surprising that the most prevalent chemicals in Table 1 also appear in the 20 most common two-way (Table 5) and three-way chemical combinations (Table 6), with the notable exceptions of octinoxate and bucinal. The most common ingredient combinations involve the paraben, glycol ether, and natural fragrance classes. Prioritization can be further improved by taking product usage patterns and likely absorption into account; for example, by accounting for differences between products that are used several times per day or products that remain on the skin (as opposed to being rinsed off after application) or products that contact mucosa rather than the hair, etc.

The analysis thus far considers only chemical combinations that occur in the same product. However, consumer products are often used in combination. Consider a typical morning regime (toothpaste, body wash, shampoo, conditioner, deodorant, and lotion) where the percentages of products that contain at least one target chemical make cumulative exposure from different products likely: 12% of toothpastes, 33.4% of body washes, 43.9% of shampoos, 58.4% of conditioners, 12.3% of deodorants, and 66.5% of lotions in our database contain at least one of the target chemicals (Table 4). Female consumers who use cosmetics have an increased risk of cumulative exposure due to the high percentage of target chemicals in eye (66.8%) and lip (42.3%) makeup and foundation (72.3%). Consumers are likely to use more than one product in a day, so the estimates reported here should be considered as a lower bound for cumulative exposure to the target chemicals. Consumers can be exposed to seven or more target chemicals in a single product. Of the 20 products with at least seven chemicals, 8 are lotions or moisturizers, 7 are hair styling products, 2 are shampoos, 1 is a body wash, one is foundation, and the last is an unclassified personal care product (data not shown). It is difficult to estimate the actual levels of exposure based on ingredient lists because regulations (21 CFR 701.3(d)) under the FPLA only require specific concentrations to be provided for pharmacologically active ingredients. The type of exposure also needs to be considered. For example, a product containing a large amount of one chemical that is applied to the skin and left on after application might lead to a greater level of exposure than a product containing multiple chemicals that is rinsed off after use.

## Conclusions

We introduced an informatics approach to aid exposure-based prioritization of near-field chemicals for risk assessment. We compiled a database from public sources to study the distribution and prevalence of 55 chemicals in consumer products that have been classified as potential EDCs or that have been associated with asthma in observational studies. The presence of these particular chemicals in consumer products was recently studied by gas chromatography-mass spectrometry (GCMS) (Dodson et al. 2012). Our database reveals the prevalence of these chemicals as well as their most common two-way (Table 5 and Excel File Table S1), three-way (Table 6 and Excel File Table S2), and higher-order combinations (Excel File Tables S3–S5). Specifically, the following combinations co-occur often in consumer products (number of products in parentheses): methyl paraben and 2-phenoxyethanol (1,872); linalool and limonene (1,850); methyl paraben and ethyl paraben (1,329); ethyl paraben and 2-phenoxyethanol (1,081); methyl paraben, ethyl paraben, and 2-phenoxyethanol (1,059). Readers can search for other combinations interactively at https://uiuc-gslis-blake.shinyapps.io/App-ChemComboBrowser.

Our results show that chemical synonymy can obscure the presence of potentially harmful ingredients. The target chemicals in this study appear under different names on product labels. Some of these chemical synonyms are benign-sounding extracts and oils that may alter a consumer’s risk perception.

The advantage of the informatics approach is that a much larger sample can be explored than in a GCMS analysis. Our sample contains 38,975 consumer products compared to only 213 in the GCMS analysis. Consequently, the target chemicals were detected in more products and across a broader range of product categories, including some that were negative in the GCMS analysis. However, our approach is limited by the availability of product labels and their degree of completeness. Ingredients that are not listed on the product label cannot be detected by the informatics approach. In contrast, GCMS can detect chemicals that are not listed on product labels (e.g., phthalate contaminants leached from product packaging and fragrance/flavor chemicals simply listed as generic fragrance or flavor). Therefore, these approaches should be considered complementary. Prevalent combinations from either approach provide a basis for prioritizing the chemical mixtures that should be further tested in order to determine if their cumulative toxicity is independent (additive), synergistic, or antagonistic.

***Editor’s Note:** In the Advance Publication, the title of the article was incorrect. The correct title is “An Informatics Approach to Evaluating Combined Chemical Exposures from Consumer Products: A Case Study of Asthma-Associated Chemicals and Potential Endocrine Disruptors.” The correction is included in this article. The authors regret this error.*

## Supplemental Material

(851 KB) PDF

(38 KB) ZIP
