# Supplementary material for: An Informatics Approach to Evaluating Combined Chemical Exposures from Consumer Products: A Case Study of Asthma-Associated Chemicals and Potential Endocrine Disruptors
Source: Environ Health Perspect. 2016 Mar 8;124(8):1155–65. doi: 10.1289/ehp.1510529 (PMC4977060; doi:10.1289/ehp.1510529)
Supplement: (851 KB) PDF [file ehp.1510529.s001.acco.pdf]

Environ Health Perspect
DOI: 10.1289/ehp.1510529

Note to readers with disabilities: *EHP* strives to ensure that all journal content is accessible to all readers. However, some figures and Supplemental Material published in *EHP* articles may not conform to [508 standards](http://ehp.niehs.nih.gov/accessibility/) due to the complexity of the information being presented. If you need assistance accessing journal content, please contact ehp508@niehs.nih.gov. Our staff will work with you to assess and meet your accessibility needs within 3 working days.

Supplemental Material (Database Methods)

An Informatics Approach to Evaluating Combined Chemical Exposures from Consumer Products: A Case Study of Asthma-Associated Chemicals and Potential Endocrine Disruptors

Henry A. Gabb and Catherine Blake

Table of Contents

Construct a Consumer Products Database Using Data from Online Sources
- Scrape Product Information from Online Retailers
  - Verify that Scraping is Allowed
  - Robotic Scraper
  - Extract the Requisite Information from the Raw HTML
- Remove Duplicate Products
- Load the Product Data into a Structured Database

Process the Chemical Dictionaries

Match Ingredient Names to PubChem and UMLS Synonyms

References

Additional files
- Supplemental Code and Data Zip File
  - Supplemental Code and Data Index
  - Excel File Tables S1-S5

Excel File Table S1. Two-way chemical combinations

Excel File Table S2. Three-way chemical combinations

Excel File Table S3. Four-way chemical combinations

Excel File Table S4. Five-way chemical combinations

Excel File Table S5**.** Six-way chemical combinations

Construct a Consumer Products Database Using Data from Online Sources

Scrape Product Information from Online Retailers

Verify that Scraping is Allowed

After confirming that data collection was consistent with the retailer's terms of use and that robotic scraping was not prohibited, consumer product data were collected from Drugstore.com. Drugstore.com's terms of use state:

> *"You agree that your use of robots, spiders, crawlers, wanderers, Web agents and other such automated processes on the Site will be Standard for Robot Exclusion (SRE) - compliant robots ("robots") and when connecting to the Site, prior to downloading or indexing any pages on the Site, such robots will immediately visit http://www.drugstore.com/robots.txt ("the robots.txt file"). You understand that the robots.txt file is the only means by which robots are authorized to access the Site. … You agree not to reproduce, duplicate, copy, sell, resell or exploit for any commercial purposes, any portion of the Site…"*

Scraping is allowed as long as robots comply with the rules in their robots.txt file and scraped data are not redistributed or used for commercial purposes. The robots.txt file provides a sitemap to help robot scrapers navigate the site, a list of disallowed branches where scrapers should not go, and a minimum crawl delay to avoid overwhelming the server with HTTP requests:

```
Sitemap: http://www.drugstore.com/Sitemaps/0/default.xml
User-agent: *
Disallow: /cart.asp
Disallow: /list.asp
Disallow: /onorder.asp
Disallow: /checkout/
Disallow: /user/
Disallow: /products/email_product.asp
Disallow: /products/writereview.asp
Disallow: /la/account/
Disallow: /la/order/
Disallow: /templates/HIPAA/info.asp
Disallow: /affiliate/content.asp
Disallow: /shoppingbag.asp
Disallow: /checkout/default.asp
Disallow: /popups/largerphoto/default.asp
Disallow: /pricing.asp
Disallow: /LookAheadSuggestions.aspx
Disallow: /templates/stdplist/default.asp
Disallow: /templates/stdcat/default.asp
Disallow: /templates/evgrndept/default.asp
```

```
Disallow: /templates/events/circular.asp
Disallow: /4213/edh
User-agent: adidxbot
Crawl-Delay: 1
```

Robotic Scraper

The robotic scraper used for this project consists of approximately 130 lines of Java code. It uses the XPath extensions to traverse a retailer's published sitemap, and the Apache HttpClient (version 3.1) to request product webpages. Note that HttpClient is no longer supported. Its functionality has been incorporated into Apache HttpComponents so new development should use this package or some other supported HTTP client (e.g., Jsoup, BeautifulSoup, cURL).

Drugstore.com was scraped in April 2014. Scraping was done on an HP SL390G7 server with two 2.66GHz Intel Xeon X5650 processors and 96GB memory. The operating system was Scientific Linux 6.1 (Linux 2.6.32 kernel). Scraping is network-limited rather than compute- or memory-limited so a powerful server with specialized hardware is not necessary. A reliable network connection and sufficient disk space are more important. Scraping Drugstore.com took approximately three days at a two-second crawl delay. Their robot exclusion protocol specified a one-second crawl delay but this was doubled to put less strain on their servers.

Extract the Requisite Information from the Raw HTML

Brand and product names, ingredient list, and product category are needed for this analysis. This information is available on most Drugstore.com product pages and can be extracted from the raw HTML retrieved by the robot scraper. This is done by finding tags that consistently mark the desired information across a given retail site. For example, the "TblProdForkIngredients" tag indicates the location of the product ingredient list in Drugstore.com product pages.

The first occurrence of the "s.prop5" and "<title>" tags indicate the brand and product names, respectively, and the "home<" tag indicates the retail hierarchy for product categorization (e.g., home → personal care → oral care → mouthwash). These tags vary by retailer but once identified are consistent and reliable across a given retailer's product pages. Frequent spot checks of random samples are used to refine each stage of data processing.

Validation of brand and product names was performed by manual inspection of 100 randomly selected products to confirm that the necessary data was correctly extracted from the raw HTML. Accuracy was 100% (i.e., every brand and product name in the sample was correct).

Category assignments were similarly validated using a random sample of 100 products. Accuracy was high (96%). Of the four incorrectly categorized products, one was due to an error in the retail hierarchy; specifically, an eyeliner product was incorrectly placed in the lip liner branch of the sitemap. The rest were due to ambiguities in category mapping. For example, one of the incorrect assignments was a topical medication in a relatively sparse branch of the retail

hierarchy: medicine & health → pain & fever relief → shop by active ingredient → natural ingredients. The most specific level of the retail hierarchy that maps to one of our product categories is "pain & fever relief" so it used to make the assignment, as stated in the article. In our categorization scheme, "pain & fever relief" maps to oral medications because most products in this category are oral medications.

A combination of Python (version 2.7.3), regular expressions, grep, and the html2text utility were used to process the raw HTML product pages. Extracting the brand names, product names, and product categories was straightforward but extracting the ingredients required more finesse because there is no standard format for ingredient lists. Most product labels provide a simple, comma-delimited list of ingredients. However, some lists contain non-ingredient text, active concentrations, and parenthetical information that may or may not be useful, e.g.:

```
active ingredients: avobenzone - 2 % (sunscreen),
homosalate (15%), octisalate (5%) (sunscreen), oxybenzone -
4 % (sunscreen) inactive ingredients: alcohol denat,
acrylates, octylacrylamide, glycerin, aloe barbadensis leaf
extract, tocopherol (vitamin e), cocos nucifera oil
(coconut), mineral oil, fragrance
```

Simply processing this string as a comma-delimited list will result in noisy ingredient names that are more difficult to match to chemicals. However, patterns in such strings inform a multistep text processing algorithm that yields a clean list of ingredients for most product label formats.

Step 1: Remove "`active ingredients:␣`" (the ␣ symbol denotes a single space) and replace "`␣inactive ingredients:␣`" with a comma.

```
avobenzone - 2 % (sunscreen), homosalate (15%), octisalate
(5%) (sunscreen), oxybenzone - 4 % (sunscreen),alcohol
denat, acrylates, octylacrylamide, glycerin, aloe
barbadensis leaf extract, tocopherol (vitamin e), cocos
nucifera oil (coconut), mineral oil, fragrance
```

Step 2: Parse the comma-delimited list with the following regular expression to get a preliminary list of ingredient strings:

```
Regex = ((?:(?:[^,]+? +?\(.+?\))+(?:[^,]+)?)|(?:[^,]+))
```

```
avobenzone - 2 % (sunscreen)
homosalate (15%)
octisalate (5%) (sunscreen)
oxybenzone - 4 % (sunscreen)
```

```
alcohol denat
acrylates
octylacrylamide
glycerin
aloe barbadensis leaf extract
tocopherol (vitamin e)
cocos nucifera oil (coconut)
mineral oil
fragrance
```

Step 3: Product labels often contain extraneous text like “usp”, “denat” or “denatured”, “certified organic”, “contains less than”, etc. so a list of the most common non-ingredient phrases was compiled. Such text is removed in this step.

Step 4: Extract active concentrations from the ingredient strings using the regular expression below. Note that active concentrations are specified in percentages, milligrams, or units. Active concentrations are not used in the present analysis but they are retained for future use.

```
Regex = ([0-9\.|\,0-9]*\s?)(%|mg|units)
```

```
avobenzone - (sunscreen)
homosalate
octisalate (sunscreen)
oxybenzone - (sunscreen)
alcohol
acrylates
octylacrylamide
glycerin
aloe barbadensis leaf extract
tocopherol (vitamin e)
cocos nucifera oil (coconut)
mineral oil
fragrance
```

Step 5: Extract parenthetical text using the regular expression below. Parenthetical text often contains information that can help identify chemical ingredients (e.g., “vitamin e” in this

example) so it is retained. Any leftover trailing punctuation is also removed in this step to yield a final, clean list of ingredient names.

```
Regex = \(([^)]+)\)
```

```
avobenzone
homosalate
octisalate
oxybenzone
alcohol
acrylates
octylacrylamide
glycerin
aloe barbadensis leaf extract
tocopherol
cocos nucifera oil
mineral oil
fragrance
```

The ingredient string processing algorithm was validated by randomly selecting 100 products for manual inspection. Parsed ingredient lists were compared to the raw ingredient strings to confirm that ingredient names and accompanying parenthetical text are correctly extracted. Of the 1587 ingredients in this sample, 1547 (97%) were correctly extracted. Of the 40 incorrectly extracted ingredients, 24 were slash-delimited polymers, fatty acids, or mixtures (e.g.: styrene/acrylates copolymer, acrylates/c10 30 alkyl acrylate crosspolymer, cetyl peg/ppg-10/1 dimethicone, caprylic/capric triglyceride, pvm/ma copolymer). The ingredient string processing algorithm was not modified to handle these types of ingredients because they are not the focus of the present analysis and because it is unclear how they should be parsed. Missing commas in the ingredient list caused the remaining 16 incorrectly parsed ingredients.

Remove Duplicate Products

Duplicate products can appear in the database for several reasons. The same product can appear in different branches of a retail sitemap. The same product may be sold in different sizes. In future, as more retail sites are scraped and added to the database, product inventories may overlap, leading to duplicate entries. Pruning duplicates is necessary to get accurate counts of products and ingredients, but identifying duplicate products is not always as straightforward as

matching product names under the same brand because typographical errors and differences in punctuation can mask duplicates, e.g.:

```
chcocolate banana jr. organic granola bar
chocolate  banana jr  organic granola bar
```

Unfortunately, digital text contains typographical errors just like printed text. If these two products have identical brands and ingredient lists, they are likely the same product scraped from different locations. Alternative word orders in product names can also mask duplicate products, e.g.:

```
chocolate cherry jr. organic granola bar

organic granola bars, chocolate cherry
```

It is harder to identify these two products as duplicates because the words, word order, and punctuation are different. However, if they also have identical brands and ingredient lists, they are likely different representations of the same product. Applying a spelling checker to fix typographical errors, removing punctuation, and doing string matching on the product names will find many duplicate products but it will not find duplicates when the word order of the product names differ. Dice's coefficient (Dice 1945) is a better way to compare product names in this case:

$$Dice\ coef. = \frac{2|S_1 \cap S_2|}{|S_1| + |S_2|}\ where\ S_N\ is\ the\ set\ of\ character\ bigrams\ in\ string\ N$$

If two product names have a high Dice coefficient, they are not necessarily the same product because formulations change. Their ingredient lists must still be compared. Labeling regulations dictate that ingredients be listed in descending order of predominance so word order matters when comparing ingredient lists. Therefore, Levenshtein ratio (Navarro 2001) is a better way to measure ingredient list similarity. It is computed as follows:

$$Levenshtein\ ratio = \frac{length(S_1) + length(S_2) - ED(S_1, S_2)}{length(S_1) + length(S_2)}\ where\ ED\ is\ the\ edit\ distance\ between\ strings\ S_1\ and\ S_2$$

Edit distance (Navarro 2001) was computed using the edit_distance function in the Natural Language Toolkit. The algorithm to find duplicate products is as follows:

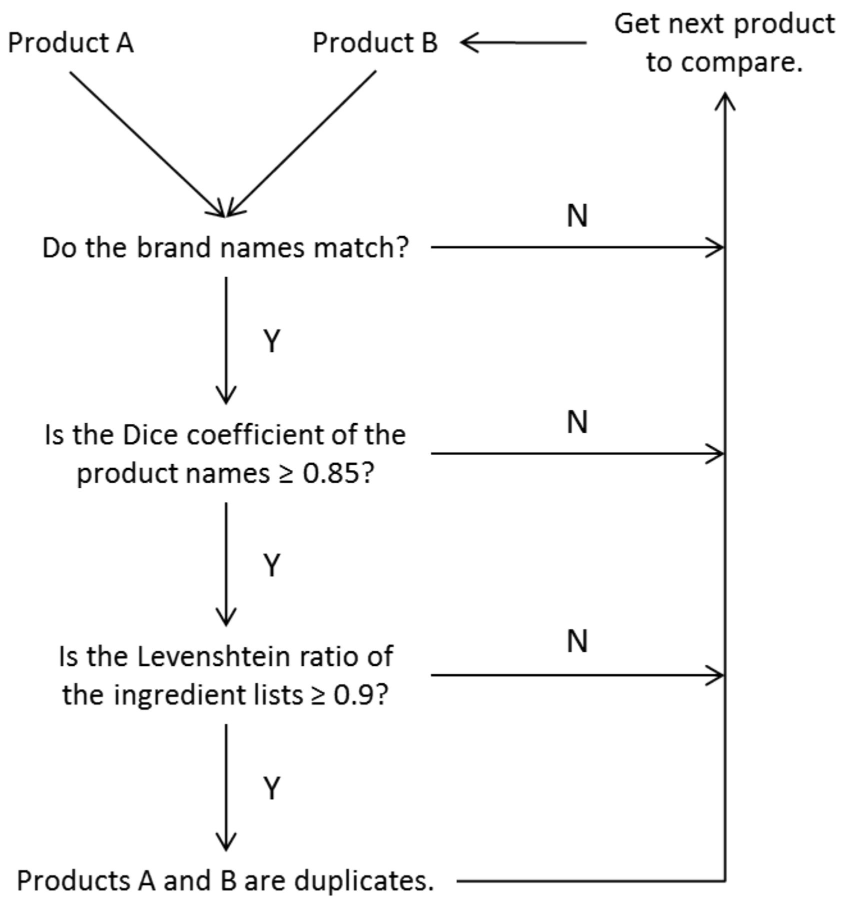

The algorithm was tuned and validated using a manually curated sample. A random sample is unlikely to contain duplicate products so ten brands with ten products each were selected and manually analyzed for duplicates. The sample contained 89 distinct and 11 duplicate products. Dice coefficient and Levenshtein ratio thresholds of 0.85 and 0.9, respectively, gave the best results, correctly identifying 9 out of 11 duplicates with no false positives.

Load the Product Data into a Structured Database

The final processed data are loaded into a structured database, in this case Oracle Database 11g Enterprise Edition (release 11.2.0.1, 64-bit production build). The following screenshot shows an example product (Biotene Oral Balance, Dry Mouth Moisturizing Gel) as it appears in the database:

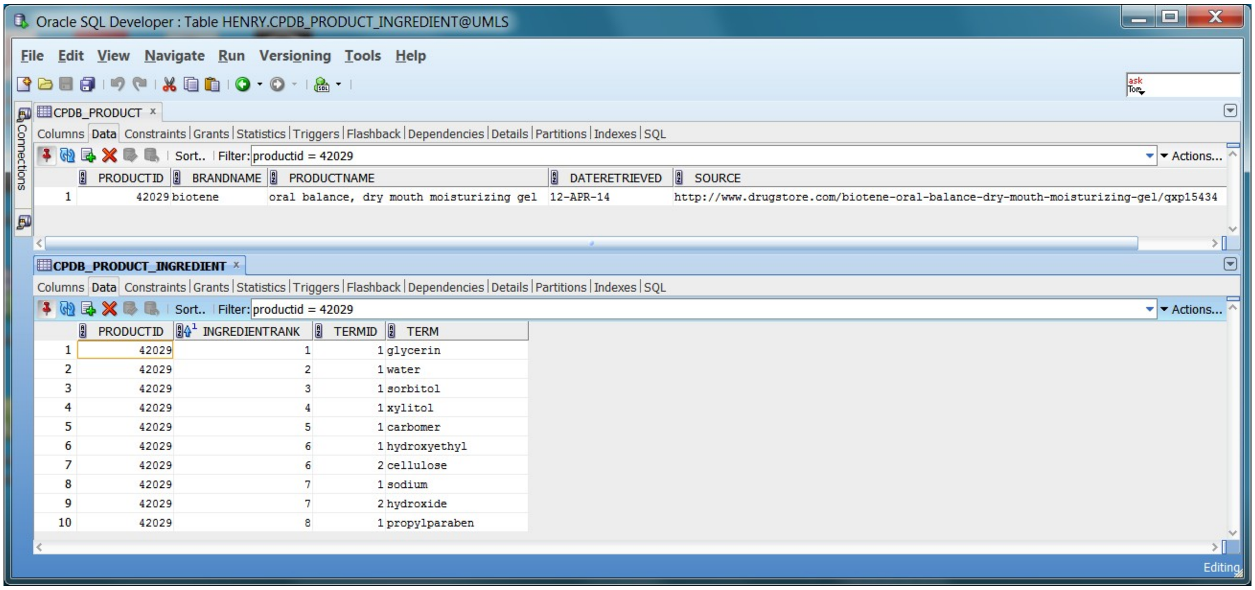

Each product is assigned a unique ID that is the primary key to access any data related to the product. The product data for the present analysis reside in two tables: one for the product details (CPDB_PRODUCT) and the other for ingredients (CPDB_PRODUCT_INGREDIENT). Other tables hold active concentrations, parenthetical information from the ingredient lists, size and price information, and textual information pertaining to the product, but they are not used in the present analysis. Note that the retrieval date (the DATERETRIEVED field) of each product is stored to help track reformulations of the same product. The order of ingredients on the product label (the INGREDIENTRANK field) is also stored because it can indicate relative predominance in the formulation. Finally, multiword ingredients (hydroxyethyl cellulose and sodium hydroxide in the example) are split into separate records (see the TERMID field) to facilitate matching with the chemical dictionaries chemicals.

Process the Chemical Dictionaries

The PubChem dictionary (Kim et al. 2016) can be downloaded from the National Center for Biotechnology Information at the National Library of Medicine: ftp://ftp.ncbi.nlm.nih.gov/pubchem/compound/extras/cid-synonym-filtered.gz. It contains approximately 39 million unique Compound Identifiers (CID) and 150 million synonyms. Some preprocessing was required to optimize name matching. Our transformations are similar to those applied to other chemical dictionaries and chemistry text processing applications (Hettne et al. 2009; McCray et al. 2001; Rogers and Aronson 2008; Schwartz and Hearst 2003). First, each synonym is converted to lowercase. Second, the long and abbreviated forms of a synonym (e.g., “acetyl hexamethyl tetralin (ahtn)”) are separated into two synonyms. Third, syntactic inversion is performed on synonyms that contain a comma followed by a space. For example, acetyl hexamethyl tetralin has a synonym “ethanone, 1-(5,6,7,8-tetrahydro-3,5,5,6,8,8-hexamethyl-2-naphthalenyl)-” that is inverted to yield an additional synonym, “1-(5,6,7,8-tetrahydro-

3,5,5,6,8,8-hexamethyl-2-naphthalenyl)-ethanone.” Finally, each synonym is split on whitespace to obtain a list of terms that are loaded into the database.

The UMLS (Humphreys and Lindberg 1993; Humphreys et al. 1998) is comprised of three components, the SPECIALIST lexicon, semantic network, and a metathesaurus that aligns the content of 170 different independently maintained controlled vocabularies: https://www.nlm.nih.gov/research/umls/knowledge_sources/metathesaurus/release/notes.html. The terms in these vocabularies are mapped to Concept Unique Identifiers (CUI). The UMLS can also be downloaded from the National Library of Medicine: http://www.nlm.nih.gov/research/umls. Terms in the UMLS were preprocessed using a process similar to Hettne et al. (2010).

The following screenshot shows the first few PubChem synonyms of sodium hydroxide as they appear in the database:

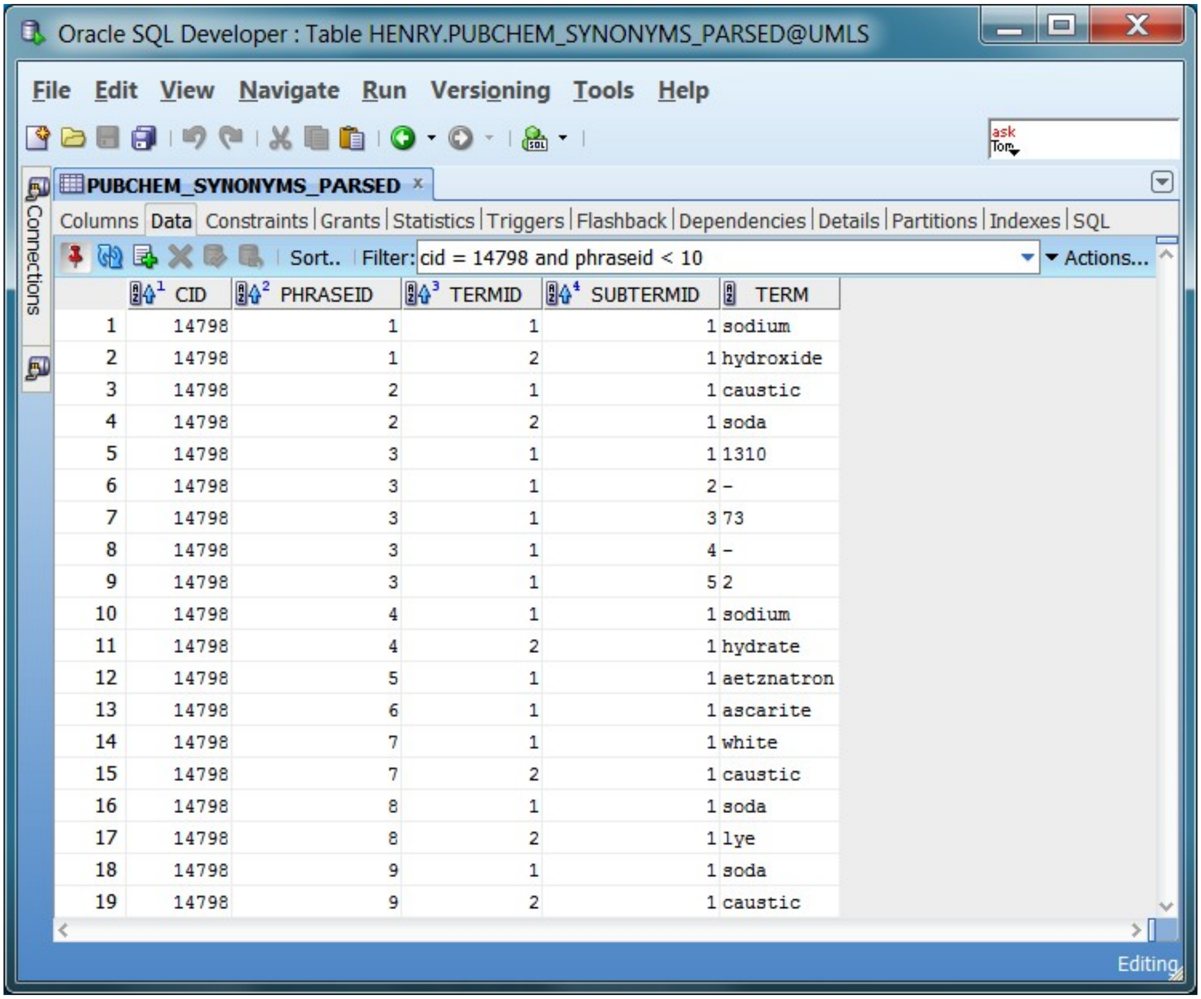

	CID	PHRASEID	TERMID	SUBTERMID	TERM
1	14798	1	1	1	sodium
2	14798	1	2	1	hydroxide
3	14798	2	1	1	caustic
4	14798	2	2	1	soda
5	14798	3	1	1	1310
6	14798	3	1	2	-
7	14798	3	1	3	73
8	14798	3	1	4	-
9	14798	3	1	5	2
10	14798	4	1	1	sodium
11	14798	4	2	1	hydrate
12	14798	5	1	1	aetznatron
13	14798	6	1	1	ascarite
14	14798	7	1	1	white
15	14798	7	2	1	caustic
16	14798	8	1	1	soda
17	14798	8	2	1	lye
18	14798	9	1	1	soda
19	14798	9	2	1	caustic

The UMLS tables have a similar structure except the unique identifier is a CUI instead of a CID. Each synonym is identified by a CID and a PHRASEID. Multiword synonyms (e.g., sodium

hydroxide) are split into individual terms that are given a TERMID. If sodium hydroxide appears in a product ingredient label, it will be mapped to CID 14798 whether it appears as sodium hydroxide, caustic soda, or soda lye.

Systematic names like 1-(5,6,7,8-tetrahydro-3,5,5,6,8,8-hexamethyl-2-naphthalenyl)-ethanone are rare in consumer product ingredient lists. Such chemicals are much more likely to be listed under a trivial name (e.g., acetyl hexamethyl tetralin) or an abbreviation (e.g., AHTN). However, to facilitate matching of systematic names, terms containing numbers and punctuation are split further into subterms (SUBTERMID).

Match Ingredient Names to PubChem and UMLS Synonyms

Ingredient names are matched to PubChem and UMLS synonyms using exact term-by-term matching. One-term ingredient names (e.g., glycerin) are simply compared to one-term PubChem synonyms and one-term UMLS concepts, two-term ingredients (e.g., sodium hydroxide) are compared to two-term synonyms/concepts, etc. If a match is found the ingredient is mapped to the CID and/or CUI. Exact matching was used for three reasons. First, as noted above, systematic names are rare in product ingredient labels so complex matching schemes are generally unnecessary. Trivial names are easily parsed into terms that can be matched exactly. Second, PubChem and UMLS entries often have dozens, sometimes hundreds, of synonyms, so a trivial name appearing in a product ingredient list is likely to be among those synonyms. Third, string matching techniques that use Dice's coefficient, edit distance, and Levenshtein ratio are prone to false positives and false negatives when dealing with chemical names. For example, "vitamin a" and "vitamin e" have a high Levenshtein ratio but are different chemicals (false positive), whereas "dimethyl ether" and "methoxymethane" have a low Levenshtein ratio but are the same chemical (false negative). A dictionary-based approach using exact term-by-term matching is therefore the best method to map an ingredient name to a chemical identifier.

References

Dice, L. R. (1945). Measures of the amount of ecologic association between species. *Ecology, 26*(3), 297-302.

Hettne, K. M., van Mulligen, E. M., Schuemie, M. J., Schijvenaars, B. J., & Kors, J. A. (2010). Rewriting and suppressing UMLS terms for improved biomedical term identification. *J Biomed Semantics, 1*(5).

Hettne, K. M., van Mulligen, E. M., Schuemie, M. J., Schijvenaars, B. J., & Kors, J. A. (2010). Rewriting and suppressing UMLS terms for improved biomedical term identification. *J Biomed Semantics, 1*(1), 5.

Humphreys, B. L., & Lindberg, D. A. (1993). The UMLS project: making the conceptual connection between users and the information they need. *Bull Med Libr Assoc, 81*(2), 170-177.

Humphreys, B. L., Lindberg, D. A., Schoolman, H. M., & Barnett, G. O. (1998). The Unified Medical Language System: An informatics research collaboration. *J Am Med Inform Assoc, 5*(1), 1-11.

Kim, S., Thiessen, P. A., Bolton, E. E., Chen, J., Fu, G., Gindulyte, A., et al. (2016). PubChem Substance and Compound databases. *Nucleic Acids Res, 44*(D1), D1202-1213.

McCray, A. T., Bodenreider, O., Malley, J. D., & Browne, A. C. (2001). Evaluating UMLS strings for natural language processing. *Proc AMIA Symp*, 448-452.

Navarro, G. (2001). A guided tour to approximate string matching. *ACM Comput Surv, 33*(1), 31-88.

Rogers, W. J., & Aronson, A. R. (2008). Filtering the UMLS metathesaurus for MetaMap. from http://skr.nlm.nih.gov/papers/references/filtering07.pdf

Schwartz, A. S., & Hearst, M. (2003). A simple algorithm for identifying abbreviation definitions in biomedical text. *Pac Symp Biocomput, 8*, 451-462.
